# Stable Bipolar Resistive Switching in Lead-Free Cs_2_AgBiBr_6_ Memristors for Neuromorphic Computing

**DOI:** 10.1021/acsomega.5c04702

**Published:** 2025-07-21

**Authors:** Fanju Zeng, Yongqian Tan, Baofei Sun, Wei Hu, Haifeng Yin, Xiaosheng Tang, Lianshuai Huang, Juan Liao, Minli Tang

**Affiliations:** † New Storage and Intelligent Computing Laboratory, School of Big Data Engineering, 117805 Kaili University, Kaili 556011, Guizhou, China; ‡ Key Laboratory of Human Brain Bank for Functions and Diseases of Department of Education of Guizhou Province, College of Basic Medical, 74628 Guizhou Medical University, Guiyang 550025, China; § Key Laboratory of Optoelectronic Technology and System of Ministry of Education, College of Optoelectronic Engineering, 47913 Chongqing University, Chongqing 400044, China; ∥ College of Optoelectronic Engineering, Chongqing University of Posts and Telecommunications, Chongqing 400065, China

## Abstract

The lead-free double-perovskite Cs_2_AgBiBr_6_, which combines nontoxicity with superior stability, has been highlighted as an ecofriendly alternative to lead-halide perovskites in photoelectric devices by recent advancements. Herein, Cs_2_AgBiBr_6_ films were successfully synthesized on a fluorine-doped tin oxide (FTO) glass substrate via a spin-coating method in a dry-air glovebox (relative humidity (RH) less than 20%), eliminating the need for antisolvent dripping or inert gas protection. The films exhibited a uniform morphology, with root-mean-square (RMS) and average (*R*_a_) roughness values of 15.4 and 11.9 nm, respectively. The fabricated Cs_2_AgBiBr_6_-based memristors demonstrated stable, repeatable bipolar resistive switching behavior with low operating voltages (+0.4 V, −0.3 V), an on/off ratio of 479, an endurance of 1000 cycles, and a retention time of 10^4^ s. Remarkably, the resistance ratio between the high-resistance state (HRS) and low-resistance state (LRS) remained stable at approximately 441 even after 30 days of ambient storage. Furthermore, the memristor mimicked biological synaptic functions, achieving an accuracy of 91.39% in machine learning simulations. These findings highlight the viability of using Cs_2_AgBiBr_6_ in both memory and computational applications, thus offering a promising pathway for developing sustainable and efficient memristor-based systems for use in neuromorphic computing and information storage.

## Graphical abstract

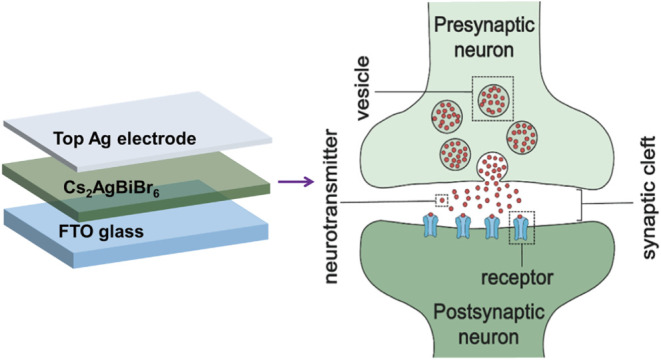

## Introduction

With the rapid development of information technology and the big data era, traditional storage technologies face significant challenges in terms of storage density, processing speed, power consumption, and durability.^1^ To meet the growing demands for future storage systems, resistive random-access memory (RRAM) has emerged as a potential solution owing to its high switching speed, low power consumption, and nonvolatile nature.^2,3^ Among the various materials explored for RRAM applications, perovskite materials have garnered considerable attention because of their excellent electrical properties, tunability, and relatively low production costs.^4–8^ In particular, perovskite-based resistive random-access memory (PRRAM) has been identified as a promising candidate for next-generation memory devices because of its unique structural and functional advantages.^9–13^

However, despite the notable advantages of halide perovskite-based materials in resistive memory devices, several challenges still impede their widespread adoption. One major issue is the relatively poor chemical and thermal stabilities of conventional lead-based perovskites.^14,15^ These materials are highly sensitive to environmental factors such as humidity, oxygen, and ultraviolet (UV) light, which can lead to degradation, reduced electrical performance, and onset of device fatigue.^16^ Such instability undermines the long-term reliability and operational sustainability of perovskite-based resistive memory devices, making environmental sensitivity a significant barrier to their practical implementation, particularly in applications that demand longevity and stable performance.^17–21^

To address these limitations, significant attention has been focused on the lead-free double-perovskite compound Cs_2_AgBiBr_6_, which inherits its structural framework from oxide double-perovskite archetypes (A_2_BB′X_6_) while incorporating mixed Ag^+^/Bi^3+^ cations at the B-site, as a strategic replacement for toxic Pb^2+^ in recent advancements.^22^ Compared with conventional lead-based perovskites, Cs_2_AgBiBr_6_ offers significant improvements in chemical stability and environmental resilience, with various advancements in neuromorphic computing and memory systems in response to environmental stressors such as humidity and oxygen. These characteristics make it a more sustainable alternative for future memory and neuromorphic computing applications.^23–29^ Cheng et al.^25^ proposed a sandwiched indium tin oxide (ITO)/Cs_2_AgBiBr_6_/Au structure for memristors, which demonstrated exceptional resilience to extreme environmental conditions, including high humidities, elevated temperatures, and radiation exposure. However, despite these advantages, Cs_2_AgBiBr_6_-based memristors still face challenges related to the high operating voltages (+3, −10 V) required for resistive switching. This elevated voltage demand poses particular issues for low-power applications, such as mobile devices, wearable electronics, and Internet of Things (IoT) devices, for which energy efficiency is a primary concern.^30,31^ Higher switching voltages increase power consumption, lead to thermal dissipation, and potentially reduce the long-term performance and reliability of devices.

In this study, Cs_2_AgBiBr_6_ films were successfully synthesized on fluorine-doped tin oxide (FTO) glass substrates via a spin-coating technique in a dry-air glovebox (with a relative humidity (RH) of less than 20%), eliminating the need for antisolvent dripping or inert gas protection. The Cs_2_AgBiBr_6_-based memristors demonstrated high memristive performance (Table S1). The resulting films exhibited a uniform surface morphology with densely packed crystal grains, confirmed by a root-mean-square (RMS) roughness of 15.4 nm and an average roughness (*R*_a_) of 11.9 nm, indicating high-quality film formation. The Cs_2_AgBiBr_6_-based memristors demonstrated stable and repeatable bipolar resistive switching (RS) behavior with low operating voltages (+0.4 V, −0.3 V), an on/off ratio of 479, an endurance of 1000 cycles, and a retention time of 10^4^ s. Notably, the resistance ratio between the high-resistance state (HRS) and low-resistance state (LRS) remained stable at 441, with minimal fluctuations observed even after 30 days of ambient storage, highlighting the excellent stability of the material in open-air conditions. Additionally, the Cs_2_AgBiBr_6_-based memristor could replicate biological synaptic functions, such as long-term potentiation (LTP) and long-term depression (LTD), which are fundamental to learning and memory. The simulation results obtained via the modified National Institute of Standards and Technology (MNIST) data set demonstrated the potential of the memristor in machine learning applications, with an accuracy of 91.39%. These promising results indicate that the Cs_2_AgBiBr_6_-based memristor is not only suitable for data storage but also holds great potential for next-generation computational applications, including neuromorphic computing and machine learning.

## Materials and Methods

### Materials

Cesium bromide (CsBr, 99.9%), silver bromide (AgBr, 99.9%), hydrogen bromide (HBr, 33 wt % in acetic acid), and bismuth bromide (BiBr_3_, ≥99.5%) were purchased from Macklin. Anhydrous DMSO (99.5%) and methyl acetate (MA, 98%) were obtained from Sigma-Aldrich. FTO substrates (1.1 mm thick, sheet resistance ∼15 Ω) were supplied by Advanced Election Technology.

### Preparation of Cs_2_AgBiBr_6_ Powder

CsBr (2.13 g, 10 mmol), BiBr_3_ (2.245 g, 5 mmol), and AgBr (0.94 g, 5 mmol) were dissolved in 50 mL of HBr with stirring at 185 °C until the solids fully dissolved. The solution was kept at 185 °C for 2 h and then cooled to room temperature. After the solution was left standing overnight, an orange powder precipitated. The precipitate was collected by filtration, washed twice with methanol, and dried under reduced pressure overnight to yield the final product.

### Preparation of Cs_2_AgBiBr_6_ Films and Device Fabrication

The FTO substrate was sequentially cleaned with deionized water, ethanol, and isopropanol via ultrasonic treatment for 30 min each. The substrate was then treated with UV ozone for 30 min. Cs_2_AgBiBr_6_ powder was dissolved in DMSO at a concentration of 0.4 M and stirred at 60 °C for 1 h to ensure uniformity. The perovskite layer was deposited on the FTO substrate via spin-coating in two steps: 1500 rpm for 50 s and 3500 rpm for 60 s. The wet films were annealed at 285 °C for 20 min on a hot plate in an air glovebox with a relative humidity below 20%. All the preparation processes were performed in a dry-air glovebox (relative humidity (RH) less than 20%), eliminating the need for inert gas protection. After the films cooled to room temperature. Silver electrodes (200 μm in diameter, approximately 120 nm thick) were deposited onto the Cs_2_AgBiBr_6_ films via thermal evaporation via a shadow mask. The deposition was carried out in a vacuum environment with a base pressure of approximately 4.0 × 10^–4^ Torr and a deposition rate of 1.0 Å/s.

### Characterization

The crystal structure of the Cs_2_AgBiBr_6_ films was analyzed via X-ray diffraction (XRD) with a Cu Kα radiation source (XRD-6100, Shimadzu, Japan). The surface and cross-sectional morphologies were examined with a scanning electron microscope (SEM, JSM-7800F, Japan). Optical absorption spectra were measured via a UV–vis spectrophotometer (UV-1800, Shimadzu, Japan). The surface roughness was assessed with an atomic force microscope (AFM, Dimension ICON, Bruker, Germany). The electrical properties of the RS memories were evaluated under ambient conditions via a Keithley 2635B semiconductor parameter analyzer.

## Results and Discussion

Lead-free double-perovskite Cs_2_AgBiBr_6_ films were directly synthesized on FTO glass substrates via a spin-coating technique conducted in a dry-air glovebox (RH less than 20%), as shown in Figure 1. The crystal structures of the Cs_2_AgBiBr_6_ films, Cs_2_AgBiBr_6_ powder, and FTO glass substrate were analyzed via X-ray diffraction (XRD), as shown in Figure 2a. The XRD patterns of the Cs_2_AgBiBr_6_ films and Cs_2_AgBiBr_6_ powder exhibit prominent Bragg peaks at 13.53° (111), 15.67° (200), 22.30° (220), 27.36° (222), 31.69° (400), 35.37° (420), 39.14° (422), and 45.50° (440). These diffraction peaks are characteristic of a cubic double-perovskite structure with *Fm*3̅*m* space group symmetry, which is consistent with previously reported literature.^32^ The Cs_2_AgBiBr_6_ films display additional peaks at 26.54, 33.76, and 37.79°, corresponding to the underlying FTO glass substrate. Importantly, the consistent presence of the characteristic Bragg peaks of the Cs_2_AgBiBr_6_ cubic double-perovskite structure in the films indicates that the crystalline integrity of the precursor powder was retained during the spin-coating process. Optical characterization of the films revealed a sharp absorption peak near 700 nm, followed by a pronounced excitonic absorption peak at approximately 580 nm (Figure 2b). This peak is attributed to excitonic transitions confined within the Cs_2_AgBiBr_6_ planar crystal structure and is consistent with previously reported observations of lead-free double perovskites.^22,33^ These results demonstrate that the employed spin-coating method successfully preserves the structural properties of Cs_2_AgBiBr_6_ during film fabrication, enabling the production of high-quality films with excellent potential for functional applications in memristor devices.

**Figure 1:**
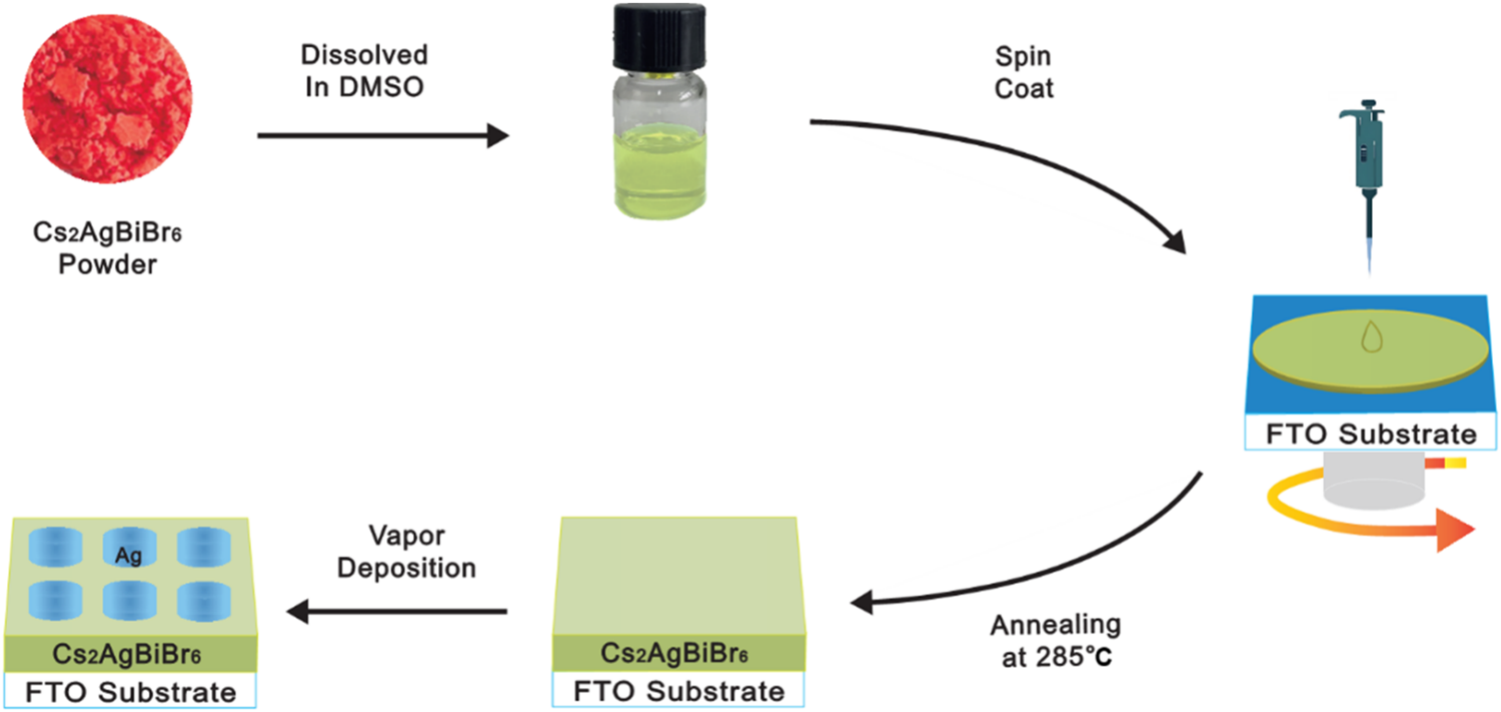
Schematic illustration of the preparation process for Cs_2_AgBiBr_6_-based memristors.

**Figure 2:**
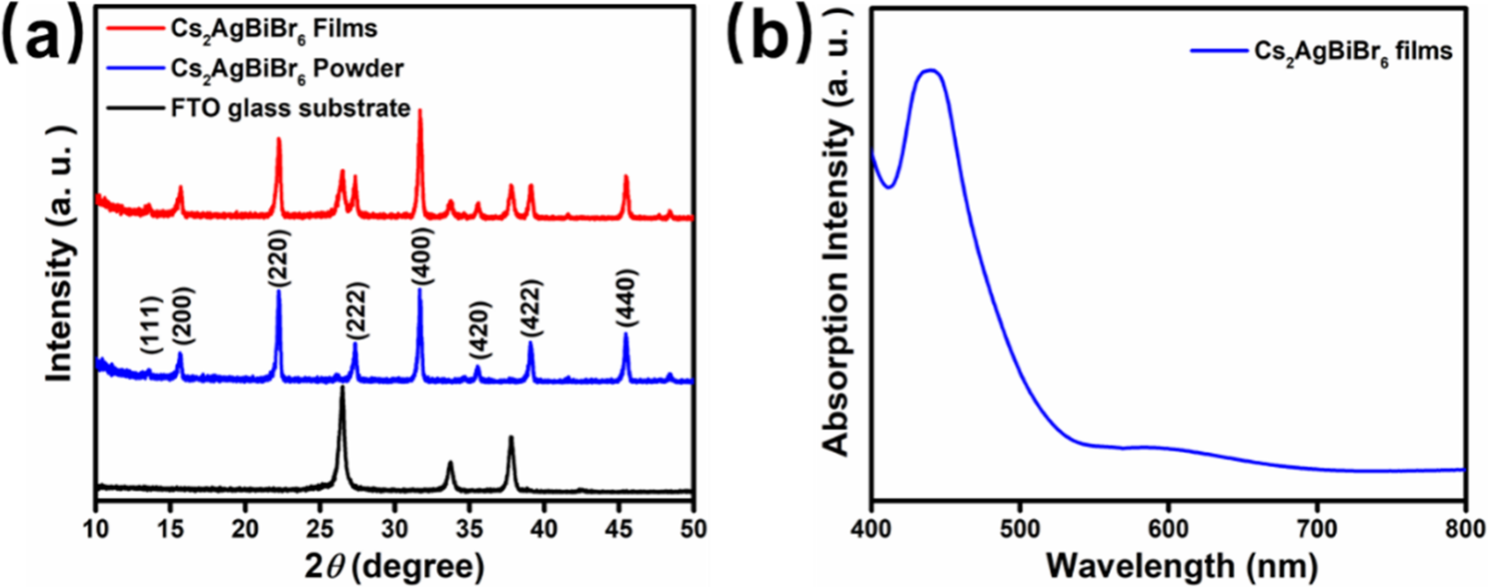
(a) XRD pattern of Cs_2_AgBiBr_6_ films, Cs_2_AgBiBr_6_ powder, and FTO glass substrate. (b) Absorption spectrum of the Cs_2_AgBiBr_6_ films.

As shown in Figure 1, the Cs_2_AgBiBr_6_ powder is a dark orange color. Scanning electron microscopy (SEM) images (Figure S1) reveals a typical octahedral crystal morphology, which is a key feature of double-perovskite structures, emphasizing its well-defined crystallinity. To comprehensively evaluate the surface and cross-sectional characteristics of the fabricated Cs_2_AgBiBr_6_ films, SEM and atomic force microscopy (AFM) were employed, providing detailed insights into the surface morphology of the films (Figure 3). SEM analysis confirmed that the Cs_2_AgBiBr_6_ films exhibited densely packed crystal grains, with no observable voids or structural defects (Figure 3a). The films have an average thickness of approximately 330 nm, with uniform deposition across the FTO glass substrate (Figure 3b). This seamless interface formation results in excellent substrate adhesion, which is critical for maintaining the mechanical and electronic integrity of device configurations. The AFM characterization (scan size: 1 × 1 μm^2^) provides quantitative metrics of the surface texture, revealing an RMS roughness of 15.4 nm and an *R*_a_ of 11.9 nm (Figure 3c,d). Uniform Cs_2_AgBiBr_6_ films were fabricated via an optimized spin-coating method performed in an air glovebox environment. A notable aspect of this fabrication protocol is its simplicity and practicality; it does not require additional steps such as antisolvent dripping or inert gas protection. This method ensures structural integrity and reproducibility while reducing the complexity and cost of the fabrication process.

**Figure 3:**
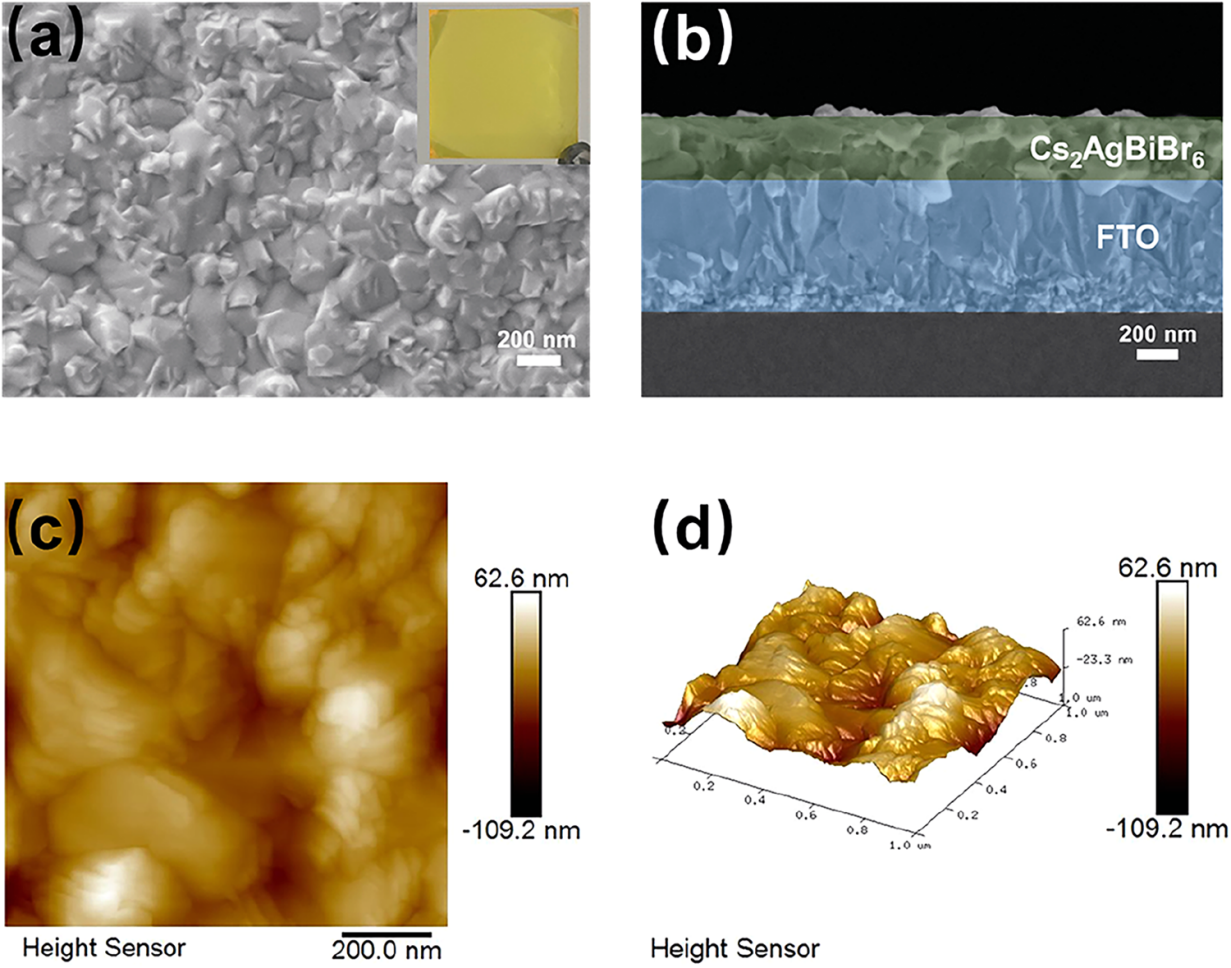
(a) Surface SEM image; the inset picture is a photograph of the Cs_2_AgBiBr_6_ films on the FTO substrate. (b) Cross-sectional SEM image, (c) surface AFM image, and (d) 3D AFM image of the Cs_2_AgBiBr_6_ films.

The schematic diagram in Figure 4a illustrates the layered structure of the Ag/Cs_2_AgBiBr_6_/FTO memristor, which consists of a functional Cs_2_AgBiBr_6_ perovskite layer, a silver (Ag) top electrode, and an FTO bottom electrode. The cross-sectional SEM image in Figure. Figure 4b provides a detailed visual representation of the structural integrity of the device. A Cs_2_AgBiBr_6_ film, approximately 330 nm thick, is uniformly deposited and positioned between the Ag electrode and the FTO glass substrate. The tight encapsulation of the Cs_2_AgBiBr_6_ layer within this sandwich-like structure ensures well-defined interfaces between the perovskite film and both electrodes, which is essential for minimizing interfacial defects that can impede charge carrier movement and hinder the RS process.

**Figure 4:**
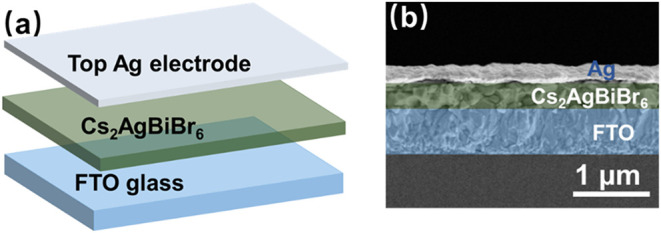
(a) Schematic of the Ag/Cs_2_AgBiBr_6_/FTO memristor vertical stack structure. (b) Cross-sectional SEM image of the memristor.

The RS behavior of the Cs_2_AgBiBr_6_-based memristor devices was thoroughly investigated by using a semiconductor parameter analyzer under ambient air conditions at room temperature. A voltage was applied to the top Ag electrode, while the bottom FTO electrode was grounded. The current–voltage (*I*–*V*) characteristics were measured, and the results are presented in Figure 5. Specifically, Figure 5a depicts the initial *I–V* curve of the Ag/Cs_2_AgBiBr_6_/FTO memristor, with distinct voltage regions corresponding to the “Set” and “Reset” operations. The device exhibits typical bipolar switching behavior, as evidenced by the pronounced hysteresis loop, which is a demonstrated feature of memristor devices.^34^ During the voltage sweep (0 V → +0.4 V → 0 V → −0.3 V → 0 V) with a compliance current of 0.5 mA, a transition occurs at approximately 0.08 V, at which point the device switches from the HRS to the LRS, and vice versa. This transition confirms the ability of the memristor to switch between these states on the basis of the applied voltage, which is a key characteristic for memory and switching applications. Figure 5b further illustrates the repeatability of the device switching behavior through multiple *I–V* curves recorded over 100 cycles. The reproducibility of these cycles affirms the stable performance of the device, which is essential for its practical use. Additionally, Figure 5c presents the endurance test results, which show that the current–voltage characteristics of the memristor remain stable over 1000 successive cycles without significant degradation (the relative fluctuations of HRS/LRS are 16.3/10.4%). The on/off resistance ratio, approximately 479, clearly indicates reliable switching between the HRS and LRS.^35^ Furthermore, the retention characteristics, as shown in Figure 5d, indicate that the resistance values in both the HRS and LRS remain virtually unchanged over a period of 10^4^ s. The absence of a forming process during the initial “Set” operation is attributed to the polycrystalline nature of the Cs_2_AgBiBr_6_ perovskite. This material inherently contains Ag ions, which facilitate the RS mechanism without the need for the initial forming step typically required in other memristor devices.^36^ To assess the device-to-device variability in the RS behavior, the *I–V* characteristics were measured at random positions across cells from eight pieces (two pieces randomly selected per batch across four batches) at a voltage of 0.08 V, as shown in Figure 6a. The relative fluctuations of the HRS range from 14.5 to 23.3%, whereas those of the HRL range from 1.3 to 11.8%. The consistent RS behavior across these cells from different batches demonstrates the uniformity of the performance of the device, reinforcing its reliability and reproducibility.

**Figure 5:**
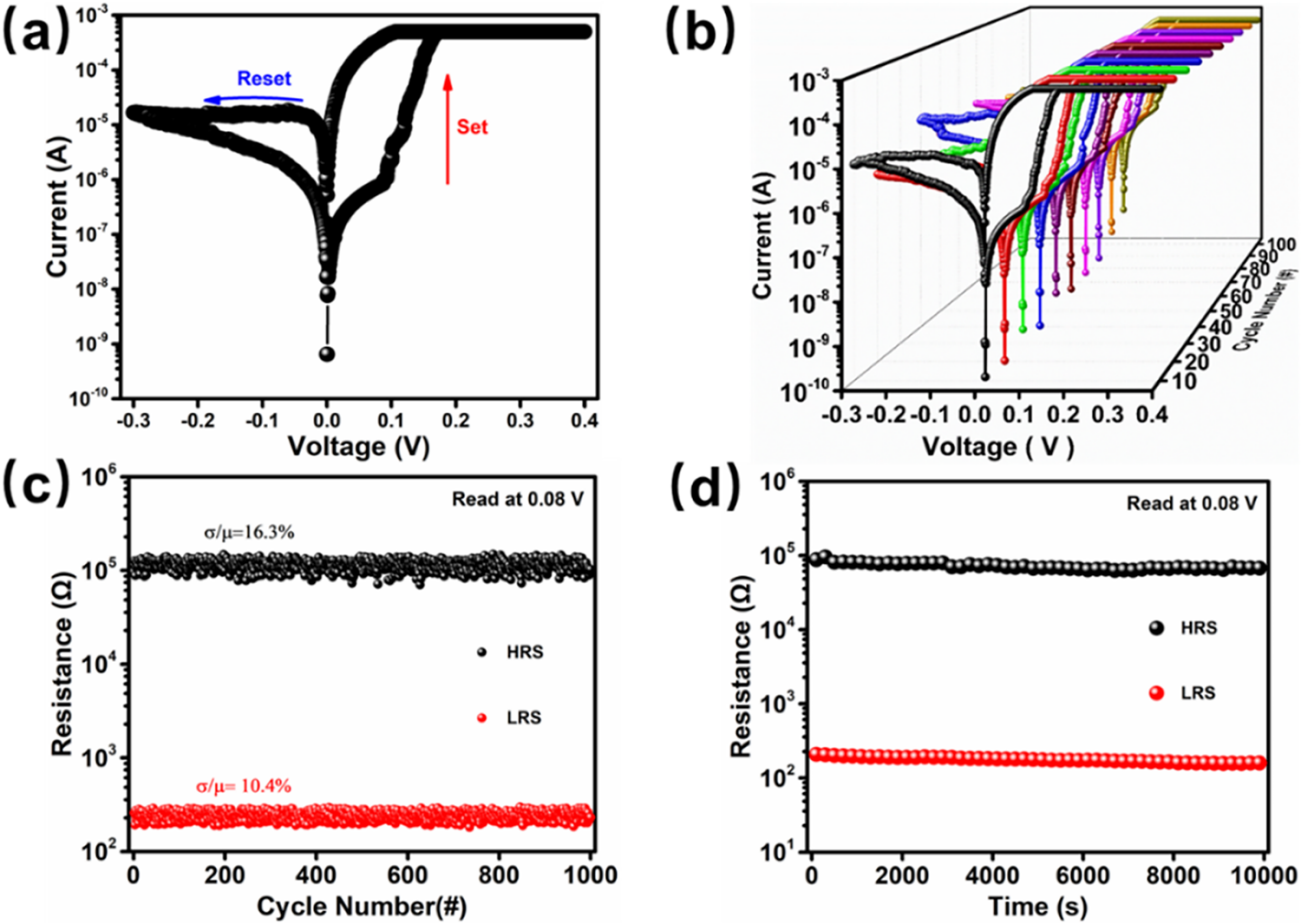
(a) Initial RS *I–V* curves, (b) cycle tests, (c) endurance, and (d) retention read at 0.08 V of the Ag/Cs_2_AgBiBr_6_/FTO memristor.

**Figure 6:**
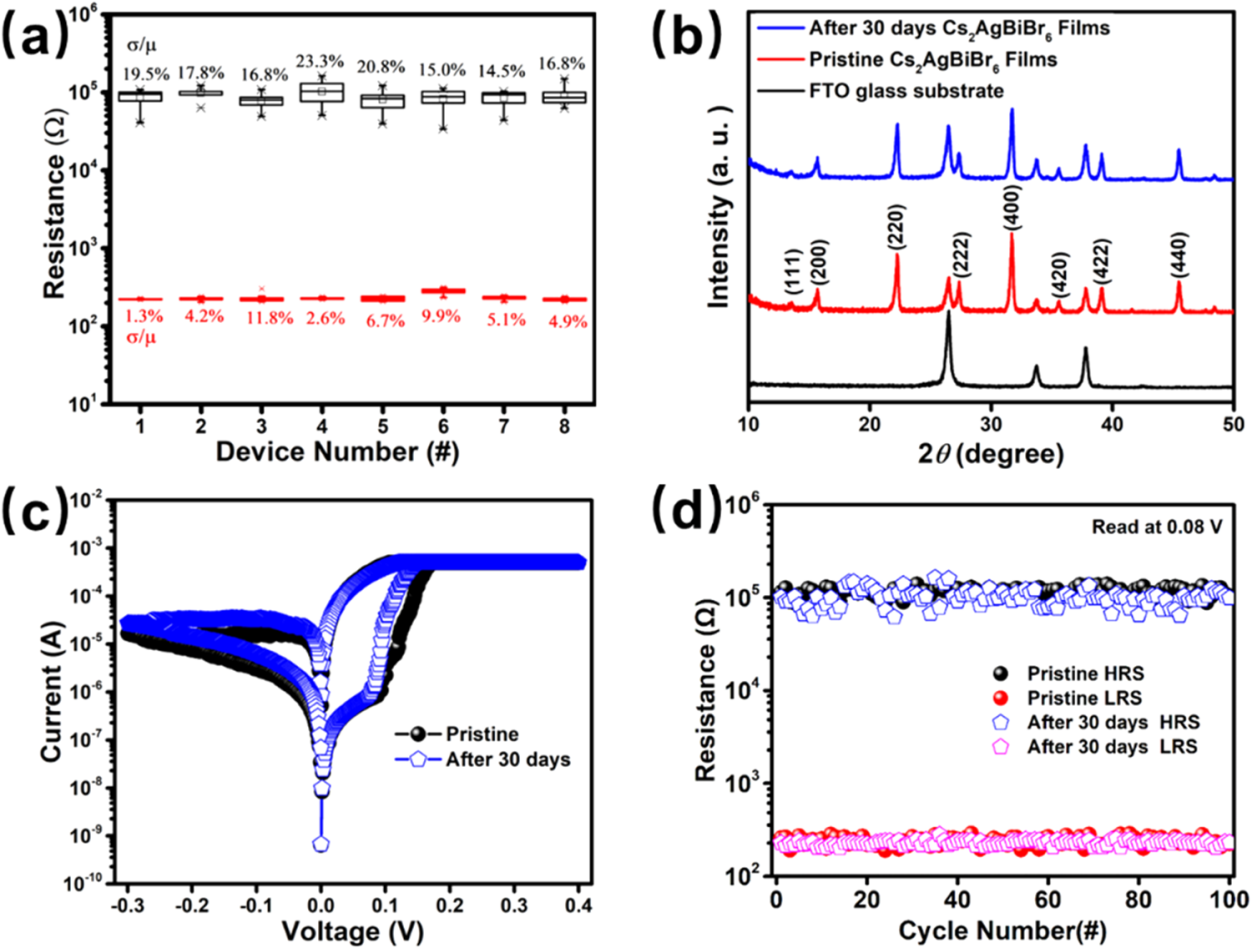
(a) HRS and LRS distributions of 8 different cells at 0.08 V (two pieces randomly selected per batch across four batches). (b) XRD patterns of the Cs_2_AgBiBr_6_ films with air exposure, pristine Cs_2_AgBiBr_6_ films, and FTO glass substrate. (c) Initial RS *I–V* curves and (d) endurance of the pristine Ag/Cs_2_AgBiBr_6_/FTO device and the Ag/Cs_2_AgBiBr_6_/FTO device after storage for 30 days.

The long-term stability was evaluated by storing the Cs_2_AgBiBr_6_ films and the corresponding devices under ambient conditions. The XRD patterns of the pristine and stored Cs_2_AgBiBr_6_ films are shown in Figure 6b shows nearly identical peak profiles after 30 days of exposure to ambient conditions, indicating that the films maintain excellent stability without the need for encapsulation layers. This result suggests that the material is resilient to environmental factors over extended periods. Additionally, the resistance ratio between the HRS and LRS remained stable at approximately 441 after 30 days of storage, with no significant fluctuations observed in either state (Figure 6d). This level of stability is critical for ensuring the long-term performance of memristor devices in nonvolatile memory applications and other switching devices, in which reliable and consistent performance is essential over time.

To investigate the carrier transport mechanisms in the Ag/Cs_2_AgBiBr_6_/FTO memristor, the conduction behavior was analyzed by replotting the *I–V* curves on a logarithmic scale, with particular emphasis on the negative-voltage region (Figure S2a,b). In this region, two distinct sections characterized by linear fittings, along with a sudden increase in current, are observed in the HRS. In the low-voltage range (0 to 0.08 V), linear ohmic behavior (*I ≈ V*) is observed, as indicated by the red line, with a slope of 1.07. This behavior suggests that conduction in this region is governed by the thermally generated free electrons, which are trapped in the Cs_2_AgBiBr_6_ film due to incomplete trap site filling. As a result, the conduction mechanism follows Ohm’s law, with the resistance being dominated by electron transport in the trap-filled state. Upon a further increase in the applied voltage, the current transitions to a square dependence on the voltage (*I ≈ V* ^2^) within the high-voltage range (0.08 to 0.09 V), with a slope of 2.17, as represented by the blue line. This transition occurs as the trap sites become fully occupied, allowing the electrons to freely move through the conduction band, which initiates the transition from the HRS to the LRS. This transition is referred to as the “set” process. Additionally, the ln­(*I*/*V*) versus *V* ^1/2^ plots for both HRS and LRS during the set process exhibit nonlinear characteristics, as demonstrated in Figure S2c,d. Furthermore, the *I–V* characteristics of the HRS current over a temperature range of 293–373 K are replotted as an Arrhenius plot (Figure S3).^37^ The conduction of the HRS current strongly relies on temperature. The activation energy (*E*_A_) for electron transport is determined to be approximately 0.12 eV. These results suggest that the conduction mechanism in the HRS regime is primarily governed by space-charge-limited current (SCLC) transport, a mechanism where the current is constrained by the density of available charge carriers rather than by the applied voltage alone, as reported in previous studies.^38,39^

The Cs_2_AgBiBr_6_ films likely contain four distinct types of vacancy defects: cesium vacancies (*V*_Cs_), silver vacancies (*V*_Ag_), bismuth vacancies (*V*_Bi_), and bromine vacancies (*V*_Br_). The calculated formation energies for cesium, silver, bismuth, and bromine vacancies are 4.74, 0.59, 3.55, and 2.56 eV, respectively.^40^ Notably, the silver vacancy has the lowest formation energy, suggesting that it is the predominant type of vacancy defect in Cs_2_AgBiBr_6_. This lower formation energy facilitates the migration of silver ions within the material. Moreover, as shown in Figure S3 and Table S2, the temperature dependence of the electrical conductivity was used to study the current transport mechanism during the RS of the fabricated devices. The LRS current is independent of temperature (293–373 K) and is linear with the applied voltage, implying metallic ohmic behavior. On the other hand, the conduction of the HRS current is highly temperature-dependent. Therefore, we propose a Ag conductive filament model for the Ag/Cs_2_AgBiBr_6_/FTO memristor (Figure S4). According to this model, when a positive bias is applied to the top Ag electrode, with the bottom FTO electrode grounded, an oxidation reaction occurs at the Ag electrode (Ag → Ag^+^ + e^–^).^41^ This reaction leads to dissolution of silver, and the resulting Ag cations migrate toward the FTO electrode under the influence of the applied electric field. Notably, the electron transfer rate is typically higher than the migration rate of Ag cations, resulting in the reduction of Ag cations when electrons reach the top electrode. When the concentration of Ag exceeds the supersaturation threshold, silver nucleation occurs, facilitating the formation of a conductive metallic filament between the Ag and FTO electrodes.^42^ When the direction of the electric field is reversed, the process is also reversed, and the Ag conductive filament is dissolved, effectively resetting the device to its initial HRS.

The Cs_2_AgBiBr_6_-based memristor exhibits stable bipolar switching behavior coupled with a low operation voltage, a high on/off ratio, excellent endurance, electrical reliability, long-term stability, and resistance to environmental degradation, making it a promising candidate for RS memory and neuromorphic computing applications. As illustrated in Figure 7a, the biological synapse model serves as an analogy, in which the presynaptic membrane, postsynaptic membrane, and neurotransmitters work in concert during neural signaling. In the Ag/Cs_2_AgBiBr_6_/FTO memristor, the top Ag and bottom FTO electrodes simulate the presynaptic and postsynaptic membranes, respectively. Like how action potentials modify synaptic weights and transmit signals in biological systems, the application of a voltage to the memristor alters its conductance. This ability to modify synaptic weights is essential for synaptic plasticity, which is a key process underlying learning and memory. The memristor conductance states, which are modulated by long-term potentiation (LTP) and long-term depression (LTD), were directly mapped to the synaptic weights in the neural network model. To explore this analogy further, we investigated the LTP and LTD characteristics of the memristor, treating it as an artificial synapse. Specifically, the memristor’s ability to switch between an HRS and an LRS emulates the change in synaptic strength that occurs during neural learning. This is key to the network̀s adaptation during training, where the conductance of the memristor is altered on the basis of the application of voltage pulses, similar to how synaptic weights are adjusted in biological systems. In Figure S5, after pulse parameter optimization, both Figures 7b and S5c demonstrate the gradual increase and decrease in conductance achieved by applying intermittent positive pulses (+0.27 V for 1 ms) and negative pulses (−0.77 V for 1 ms), respectively. Potentiation and depression processes are observed over 100 conductance states, with the conductance incrementally changing in response to pulse voltages. The maximum conductance was measured as 4.7920 × 10^–5^ S, and the minimum conductance was measured as 2.5256 × ^10–6^ S, resulting in an approximately 18.97-fold change in the conductance. In neuromorphic computing, LTP and LTD are crucial mechanisms for synaptic weight updates, mimicking biological synapse behavior. The weight update is typically modeled using equations that relate the conductance of the synapse to the number of pulses applied, reflecting the gradual nature of synaptic changes in response to stimuli. The equations for LTP and LTD used in the model are given by^43^

1 $$G_{\mathrm{LTP}}=B\left(1-e^{\left(\frac{-P}{A}\right)}\right)+G_{\min}$$

2 $$G_{\mathrm{LTD}}=-B\left(1-e^{\left(\frac{P-P_{\max}}{A}\right)}\right)+G_{\max}$$

3 $$B=\frac{G_{\max}-G_{\min}}{1-e^{\frac{{-P}_{\max}}{A}}}$$

where *G*_LTP_ and *G*_LTD_ are the conductances for LTP and LTD, respectively. *G*_max_, *G*_min_, and *P*_max_, which represent the maximum conductance, minimum conductance and maximum number of pulses required to switch the device between the minimum and maximum conductance states, respectively, are directly extracted from the experimental data. The parameter A governs the nonlinear behavior of the weight update process, with its value being either positive or negative depending on whether Long-Term Potentiation (LTP) or Long-Term Depression (LTD) is occurring. These equations describe the evolution of synaptic weight, represented by conductance, as it is modulated through a series of programming pulses. The nonlinear dynamics arise from the exponential dependence of the conductance change on the number of pulses, with the parameter *A* controlling the steepness of this exponential transition. Physically, the conductance of a synaptic device, such as a memristor, is incrementally altered with each pulse, reflecting the learning rules observed in biological synapses. The parameters *G*_min_ and *G*_max_ define the lower and upper bounds of the conductance range, corresponding to the minimum and maximum synaptic strength, respectively. The parameter *A* dictates the rate at which the synaptic strength transitions between these bounds, with higher values of *A* leading to a sharper transition. The weight update process exhibits asymmetry between LTP and LTD, with LTP and LTD occurring at different rates. This asymmetry is captured by the exponential function, where the conductance initially changes rapidly (during the potentiation or depression phase) and then saturates gradually, reflecting the physical limitations inherent in resistive switching devices. This model incorporates the nonlinear nature of synaptic weight updates, where the weight change is most pronounced in the early stages and then levels off as the device approaches its conductance limits. The equation for *B*, which is derived from the maximum and minimum conductance values (*G*_max_ and *G*_min_) and the maximum pulse number (*P*_max_), ensures that the conductance update behavior remains within realistic bounds. This formulation allows for an accurate representation of the gradual and nonlinear weight changes during both potentiation and depression, while also accommodating the specific characteristics of the resistive memory devices, such as the number of conductance states, precision, and device-to-device variability. In this work, using a MATLAB fitting script, we computed the nonlinearities for LTP and LTD, which were found to be −1.55 and −4.88, respectively. The memristor exhibits a wide range of conductance states in response to step pulses, significantly enhancing the accuracy of the system by providing uniformly spaced conductance values for hardware neural networks. In the context of neural network training, memristors play a pivotal role in updating weights. During training, the resistance of the memristor is dynamically adjusted to optimize model learning. To evaluate this behavior, we simulated the performance of the memristor in image recognition tasks via the MNIST data set through the NeuroSimV3.0 platform (Figures 7b and S6), which was developed by Yu et al.^43^ at Arizona State University. This platform offers a comprehensive simulation framework for benchmarking synaptic devices and array architectures and assessing system-level learning accuracy and hardware performance. In the simulation, a two-layer multilayer perceptron (MLP) neural network was utilized for both online learning and offline classification with the MNIST data set. The network topology consisted of 400 neurons in the input layer, 100 neurons in the hidden layer, and 10 neurons in the output layer, with learning rates of 0.4 and 0.2 for the input-to-hidden and hidden-to-output layers, respectively. The momentum method was employed for training. Figure 7d displays the recognition accuracy for handwritten digit images on the basis of LTP/LTD data under fixed pulse voltages. After 125 learning epochs, the recognition accuracy reaches 91.39%, with the correct and incorrect recognition results highlighted in the inset of Figure 7d. This high accuracy demonstrates the significant potential of Ag/Cs_2_AgBiBr_6_/FTO interfacial memristor devices in machine learning applications, particularly in image recognition tasks, highlighting their potential for real-world neuromorphic computing implementations.

**Figure 7:**
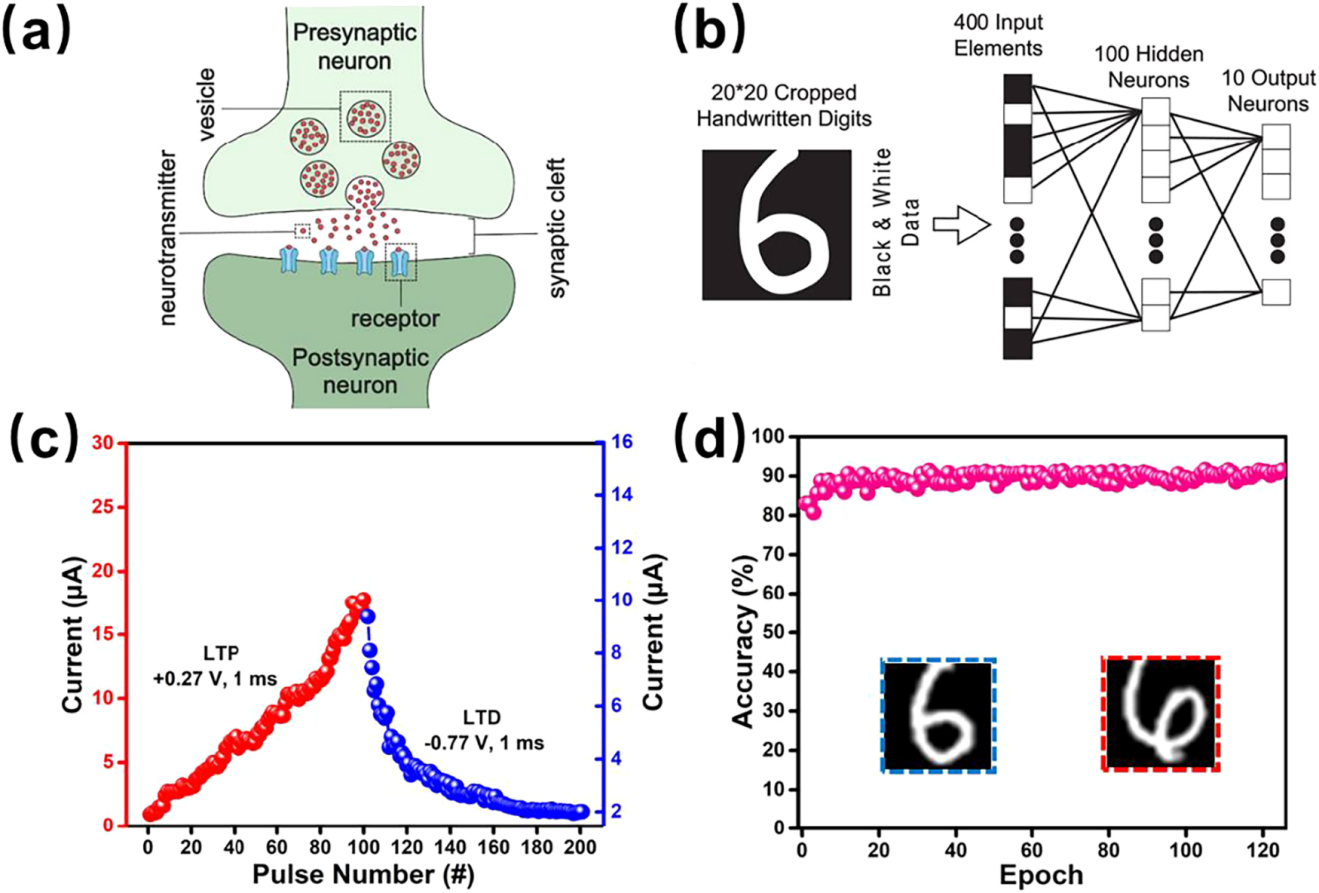
(a) Schematic depiction of synaptic signal transmission. (b) Long-term potentiation and depression. (c) Recognition accuracy for handwritten digit data. The inset shows the results of correct (in the blue box) and incorrect (in the red box) recognition of the number 6.

## Conclusions

In this study, we successfully synthesized lead-free double-perovskite Cs_2_AgBiBr_6_ films on FTO glass substrates via a spin-coating method performed in a dry-air glovebox (RH less than 20%) without the need for antisolvent dripping or inert gas protection. The resulting Ag/Cs_2_AgBiBr_6_/ITO memristor demonstrated promising performance characteristics. The Cs_2_AgBiBr_6_ films exhibited a uniform surface morphology with densely packed crystal grains, as evidenced by the RMS and Ra values of 15.4 and 11.9 nm, respectively. Notably, the Cs_2_AgBiBr_6_-based memristor displayed excellent uniformity and reliable bipolar RS behavior. The device achieved an on/off ratio of 479, an endurance of 1000 cycles, and a retention time of 10^4^ s under low operating voltages (+0.4 V, −0.3 V). Furthermore, the resistance ratio between the HRS and LRS remained stable at approximately 441, with minimal fluctuations even after 30 days of exposure to ambient air. In addition to its excellent RS performance, the device was evaluated for its ability to emulate key biological synaptic functions, such as LTP and LTD, both of which are critical for learning and memory. Simulations based on the modified MNIST data set further demonstrated the potential of the memristor for machine learning applications, achieving a recognition accuracy of 91.39%. These results demonstrate the exceptional stability of Cs_2_AgBiBr_6_-based memristors, particularly in terms of their low-voltage operation and resistance to environmental degradation, while emphasizing their promising potential for use in information storage and neuromorphic computing applications. In conclusion, while the Cs_2_AgBiBr_6_-based memristors demonstrated excellent performance at the device level, scaling up these devices into larger arrays for high-density memory applications remains an important future direction. Further research and development are required to optimize the integration of these devices into large-scale arrays, focusing on challenges such as uniformity, interconnection reliability, and overall system performance. Moreover, it is crucial to explore the compatibility of Cs_2_AgBiBr_6_-based memristors with existing CMOS technology and flexible platforms. Such integration could pave the way for cost-effective, high-performance memory solutions with enhanced scalability, potentially offering greater flexibility for various electronic and photonic applications.

## Supplementary Material



## Notes

SEM image of Cs_2_AgBiBr_6_ powder, log I–log *V* curves with fitted conduction mechanisms under positive DC sweeps, schematic illustration of the formation/rupture of the silver conducting filament under an external electric field, conductance variation, and the 2-layer MLP neural network (PDF)
