# Supplementary material for: Stable Bipolar Resistive Switching in Lead-Free Cs2AgBiBr6 Memristors for Neuromorphic Computing
Source: ACS Omega. 2025 Jul 21;10(30):33731–40. doi: 10.1021/acsomega.5c04702 (PMC12332778; doi:10.1021/acsomega.5c04702)
Supplement: Supplementary file 1 [file ao5c04702_si_001.pdf]

Supporting Information

Stable Bipolar Resistive Switching in Lead-Free $Cs_2AgBiBr_6$ Memristors for Neuromorphic Computing

Fanju Zeng[1*], Yongqian Tan[1], Baofei Sun[2*], Wei Hu[3], Haifeng Yin[1], Xiaosheng Tang[4*], Lianshuai Huang[1], Juan Liao[1], and Minli Tang[1]

1. School of Big Data Engineering, Kaili University, Guizhou Kaili, 556011, China

2. Key Laboratory of Human Brain bank for Functions and Diseases of Department of Education of Guizhou Province, College of Basic Medical, Guizhou Medical University, Guiyang 550025, China

3. College of Optoelectronic Engineering, Chongqing University of Posts and Telecommunications, Chongqing 400065, China

4. Key Laboratory of Optoelectronic Technology and System of Ministry of Education, College of Optoelectronic Engineering, Chongqing University, Chongqing 400044, China

AUTHOR INFORMATION

Corresponding Author

Fanju Zeng E-mail address: z-f-j-1@163.com

Baofei Sun E-mail address: sunbaofei@gmc.edu.cn

Xiaosheng Tang E-mail address: xstang@cqu.edu.cn

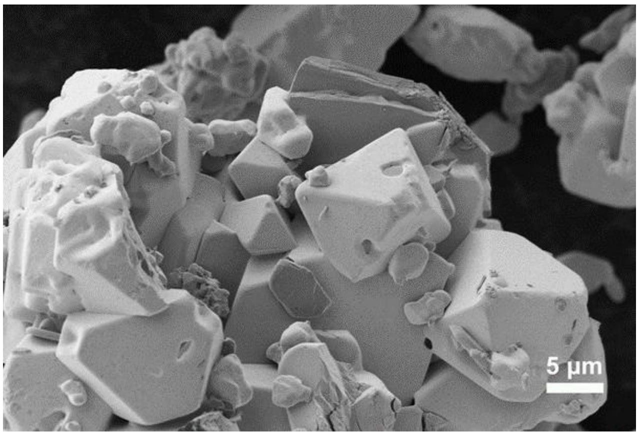

Fig. S1. The SEM image of $Cs_2AgBiBr_6$ powder.

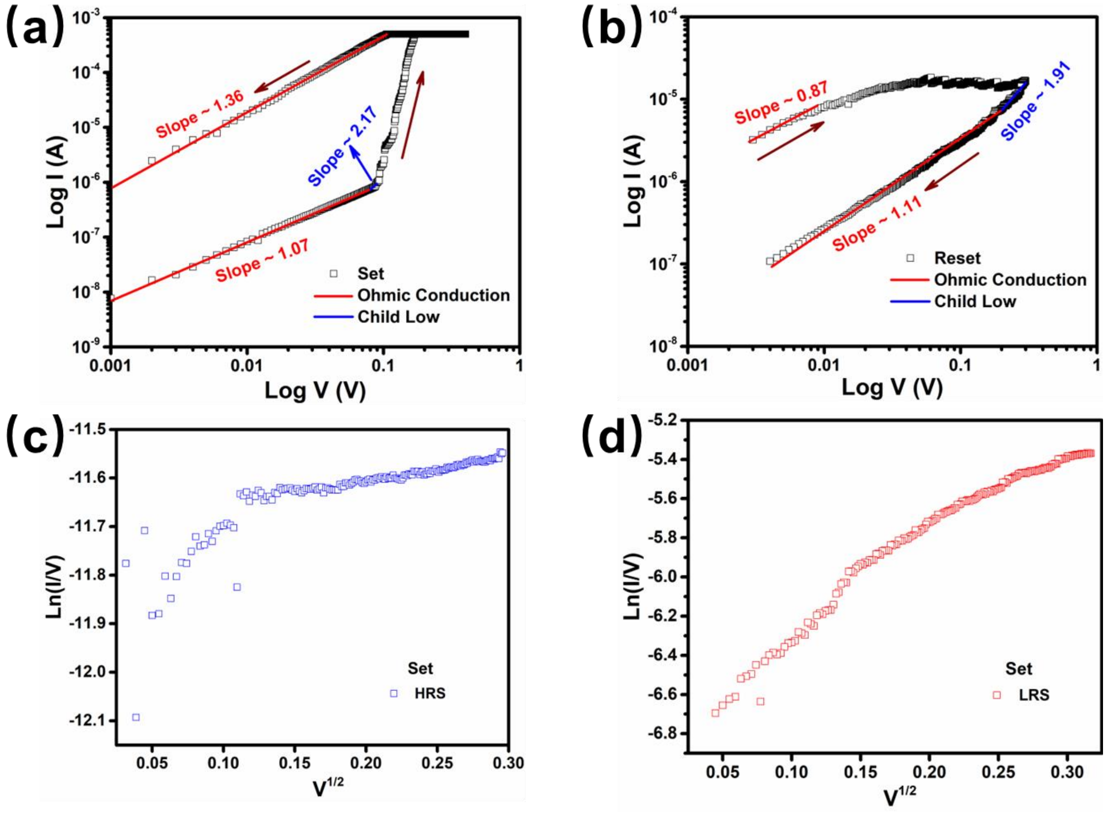

Fig. S2. Log *I*-log *V* curves with fitted conduction mechanisms under positive DC sweeps. (b) Log *I*-log *V* curves with fitted conduction mechanisms under negative DC sweeps. (c) Ln *(I/V)*- $V^{1/2}$ curves of HRS. (d) Ln *(I/V)*- $V^{1/2}$ curves of LRS in set process to analyze the conduction mechanism of $Ag/Cs_2AgBiBr_6/FTO$ memory devices.

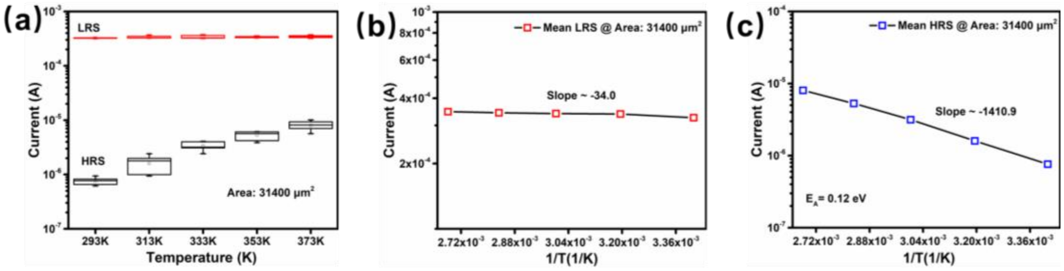

Fig. S3. (a) HRS and LRS distributions of the device at temperatures ranging from 293 to 373 K. Temperature dependence of the current for the (b) LRS and (c) HRS of the device.

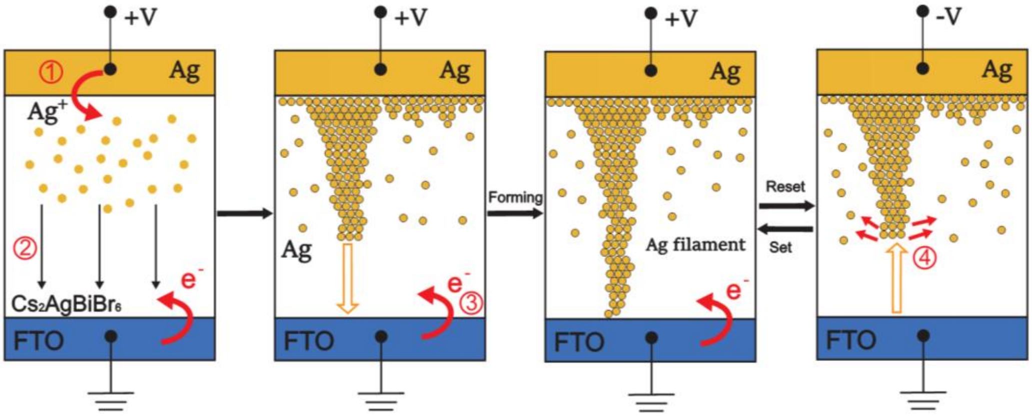

Fig. S4. Schematic illustration of the formation/rupture of the silver conducting filament under the external electric field.

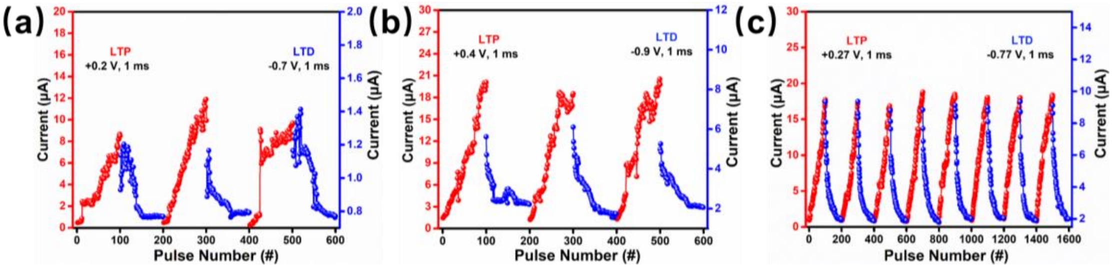

Fig. S5. Conductance variation upon consecutive potentiation and depressing pulses at different voltages: (a) +0.2 V/–0.7 V (1 ms), (b) +0.4 V/–0.9 V (1 ms), and (c) +0.27 V/–0.77 V (1 ms).

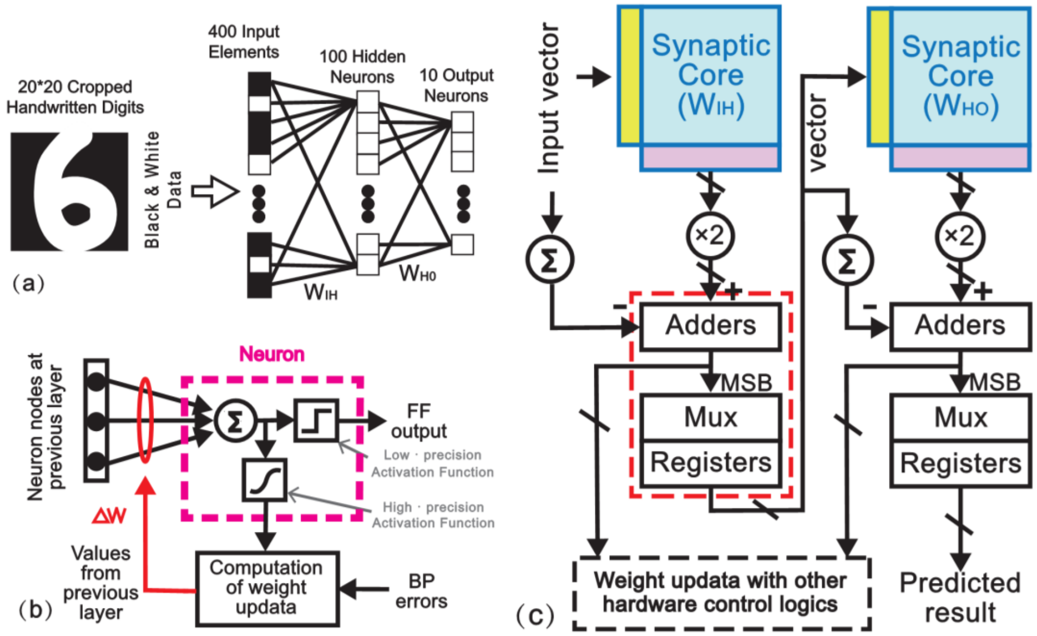

Fig. S6. (a) The 2-layer MLP neural network. (b) Schematic of a neuron node. (c) Circuit block diagram for hardware implementation of the 2-layer MLP network.

Table S1. Resistive switching performance of resistive switching memory parameters based on lead-free $Cs_2AgBiBr_6$ halide perovskites.

Memristor	Set/Reset Voltage (V)	Operation Voltage (V)	On/Off ratio	Endurance (times)
ITO/$Cs_2AgBiBr_6$/Au [1]	-3.4/2	-10/3	10^2	1000
ITO/SnO_2/$Cs_2AgBiBr_6$/NiO_x/Ag [2]	0.12/-0.7	0.7/-0.7	50	300
FTO/$Cs_2AgBiBr_6$/Ag [3]	0.14/-1.5	1.5/-1.5	15	80
ITO/$Cs_2AgBiBr_6$/Al [4]	-0.5/1.1	-3.5/3	10^3	500
ITO/$Cs_2AgBiBr_{6-x}Cl_x$/Al [5]	-0.21/1.34	-1.5/3	10^4	100
ITO/$Cs_2AgBiBr_6$/PMMA/Ag [6]	0.5/-0.5	0.5/-0.5	10	110
Au/$Cs_2AgBiBr_6$/Au [7]	-0.92/1.02	-4/4	10^7	
ITO/$Cs_2AgBiBr_6$/Ta [8]	0.04/-1	1/-1	10	100
ITO/$Cs_{2-x}Rb_xAgBiBr_6$/Ag [9]	0.1/-1	0.5/-1	9.29×10^2	100
FTO/ $Cs_2AgBiBr_6$/Ag (This work)	0.08/-0.3	0.4/-0.3	10^2	1000

Table S2. The T, 1/T, mean I_{LRS}, current density of LRS (J_{LRS}), Ln (J_{LRS}), mean I_{HRS}, current density of HRS (J_{HRS}), and Ln (J_{HRS}) were calculated from Figure S3.

T(K)	1/T (10^{-3} 1/K)	Mean I_{LRS} ($\times 10^{-4}$ A)	J_{LRS} (A/m^2)	Ln(J_{LRS})	Mean I_{HRS} ($\times 10^{-6}$ A)	J_{HRS} (A/m^2)	Ln(J_{HRS})
293	3.41	3.26	10373.66	9.25	0.76	24.21	3.19
313	3.20	3.39	10782.74	9.29	1.60	51.00	3.93
333	3.00	3.40	10843.20	9.29	3.15	100.24	4.61
353	2.83	3.43	10928.78	9.30	5.30	168.89	5.13
373	2.68	3.47	11054.35	9.31	8.04	255.94	5.54

REFERENCES

(1) Cheng, X.-F.; Qian, W.-H.; Wang, J.; Yu, C.; He, J.-H.; Li, H.; Xu, Q.-F.; Chen, D.-Y.; Li, N.-J.; Lu, J.-M. Environmentally Robust Memristor Enabled by Lead-Free Double Perovskite for High-Performance Information Storage. *Small* **2019**, 15 (49), 1905731.

(2) Zhai, S.; Gong, J.; Feng, Y.; Que, Z.; Mao, W.; He, X.; Xie, Y.; Li, X.; Chu, L. Multilevel Resistive Switching in Stable All-Inorganic n-i-p Double Perovskite Memristor. *iScience* **2023**, 26, 106461.

(3) Zeng, F. J.; Tan, Y. Q.; Hu, W.; Tang, X. S.; Yin, H. F.; Jing, T.; Huang, L. S.; Yang, Y.; Liao, J.; Zhou, C. M., Synthesis and Resistive Switching Performance of Lead-Free Double Perovskite $Cs_2AgBiBr_6$ Films. *Appl. Phys. Lett.* **2024**, 124, 162101.

(4) Tang, J.; Pan, X.; Chen, X.; Jiang, B.; Li, X.; Pan, J.; Qu, H.; Huang, Z.; Wang, P.; Duan, J.; Ma, G.; Wan, H.; Tao, L.; Zhang, J.; Wang, H. Flexible Memristor Based on Lead-Free $Cs_2AgBiBr_6$ Perovskite for Artificial Nociceptors and Information Security. *Adv. Funct. Mater.* **2025**, 35, 2412375.

(5) Sun, C.; Luo, F.; Ruan, L.; Tong, J.; Yan, L.; Zheng, Y.; Han, X.; Zhang, Y.; Zhang, X. Enhanced Memristive Performance of Double Perovskite $Cs_2AgBiBr_{6-x}Cl_x$ Devices by Chloride Doping. *ChemPlusChem* **2021**, 86, 1530–1536.

(6) Lao, J.; Xu, W.; Jiang, C.; Zhong, N.; Tian, B.; Lin, H.; Luo, C.; Travas-seidic, J.; Peng, H.; Duan, C. An Air-Stable Artificial Synapse Based on a Lead-Free Double Perovskite $Cs_2AgBiBr_6$ Film for Neuromorphic Computing. *J. Mater. Chem. C*, **2021**, 9, 5706–5712.

(7) You, Q.; Huang, F.; Fang, F.; Zhu, J.; Zheng, Y.; Fang, S.; Zhou, B.; Li, H.; Han, C.; Shi, Y. Controllable Volatile-to-Nonvolatile Memristive Switching in Single-Crystal Lead-Free Double Perovskite with Ultralow Switching Electric Field. *Sci. China Mater.* **2023**, 66, 241–248.

(8) Zhang, X.; Wang, K.; Li, Z.; Qi, J.; Li, D.; Luo, J.; Liu, J. Fabrication of High Quality Lead-Free Double Perovskite $Cs_2AgBiBr_6$ Thin Film and Its Application in Memristor with Ultralow Operation Voltage. *Nanotechnology* **2024**, 35, 195708.

(9) Jung, U.; Lim, J.; Kim, S.; Park, J. Effects of Rubidium Substitution of $Cs_{2-x}Rb_xAgBiBr_6$ Double Halide Perovskites on Resistive Switching Characteristics for Memory Applications. *J. Alloys Compd.* **2024**, 972, 172771.
